# Effect of the Addition of WO_3_ on the Structure and Luminescent Properties of ZnO-B_2_O_3_:Eu^3+^ Glass

**DOI:** 10.3390/molecules29112470

**Published:** 2024-05-24

**Authors:** Aneliya Yordanova, Lyubomir Aleksandrov, Margarita Milanova, Reni Iordanova, Petia Petrova, Nikolay Nedyalkov

**Affiliations:** 1 Institute of General and Inorganic Chemistry, Bulgarian Academy of Sciences, G. Bonchev, Str., bld. 11, 1113 Sofia, Bulgaria; a.yordanova@svr.igic.bas.bg (A.Y.); margi@svr.igic.bas.bg (M.M.); reni@svr.igic.bas.bg (R.I.); 2 Institute of Optical Materials and Technologies “Acad. Jordan Malinowski”, Bulgarian Academy of Sciences, Blvd. Akad. G. Bonchev 109, 1113 Sofia, Bulgaria; petia@iomt.bas.bg; 3 Institute of Electronics, Bulgarian Academy of Sciences, Tzarigradsko Shousse 72, 1784 Sofia, Bulgaria; nned@ie.bas.bg

**Keywords:** glasses, structure, europium, photoluminescence

## Abstract

Glasses with the compositions in mol % of 50ZnO:(50 − x)B_2_O_3_:0.5Eu_2_O_3_:xWO_3_, x = 0, 1, 3, 5 and 10 were obtained by applying the melt-quenching method and investigated by Raman spectroscopy, DSC analysis and photoluminescence (PL) spectroscopy. Raman spectra revealed that tungstate ions incorporate into the base zinc borate glass as tetrahedral [WO_4_]^2−^ groups, and octahedral [WØ_4_O_2_]^2−^ species with four bridging and two non-bridging oxygen atoms. There are also metaborate, [BØ_2_O]^−^ and pyroborate units, [B_2_O_5_]^4−^, in the glass networks. The glasses are characterized by good transmission in the visible region, at about 80%. Photoluminescence (PL) spectra evidenced that WO_3_ is an appropriate constituent for the modification of zinc borate glass structure and for enhancing the Eu^3+^ luminescent intensity. The most intense luminescence peak observed, at 612 nm, suggests that the glasses are potential materials for red emission.

## 1. Introduction

Distinct visible emission of glasses doped with rare earth (RE) ions has attracted researchers to the development of glasses for various optical devices such as lasers, upconverters, stimulated phosphors, white light-emitting diodes (WLEDs) and optical amplifiers [1]. The luminescent and absorption properties of RE ions in glasses greatly depend on the chemical composition, structure and nature of the bonds of the host glass [2].

B_2_O_3_ is a good glass former and can form glass alone with good transparency, high chemical durability, thermal stability and good rare earth ion solubility [3]. Borate-based glasses are good hosts for RE ions, having flexibility for both the size and composition of the materials [4]. In the glass network, ZnO acts as a glass former as well as modifier, which depends on its mole concentration [5]. Its presence in the glass composition shortens the time taken for the solidification of glasses during the quenching process. Glasses containing ZnO have high chemical stability and less thermal expansion. Their wide band gap and intrinsic emitting property make them promising candidates for the development of optoelectronic devices, solar energy concentrators, ultraviolet emitting lasers, and gas sensors [6]. ZnO is thermally stable and appreciably covalent in character [7]. WO_3_ is a semi-glass former with various structural units, like tetrahedral and octahedral units (WO_4_ and WO_3_) of W^6+^ and W^5+^ ions in the glass network [8]. Particularly, the addition of WO_3_ to glasses enables special features such as the enhancement of the devitrification resistance and chemical durability of the glasses and increases in the solubility of rare earth ions in the host glass network [9]. Tungsten ions are well known for their unusual influence on the optical and electrochemical properties of glasses [10,11]. Due its high polarizability, WO_3_ could contribute to a great extent to the obtaining of high-refractive-index glasses, the enhancement of non-linear optical properties and improvements in the luminescent properties of RE ions [10].

In our recent articles, we have reported about the synthesis of tungsten-modified zinc borate glasses and glass crystalline materials of compositions 50ZnO:(50 − x)B_2_O_3_:xWO_3_, x = 0, 10, 15, 20 mol % doped with different amounts of Eu_2_O_3_ (0.1; 0.5; 1; 2; 5 and 10 mol %) [12,13]. It was found that the addition of 10 mol % WO_3_, at the expense of B_2_O_3_, increases glass density, providing clear and homogeneous bulk glass samples and exhibiting a better emission performance compared to the base binary zinc borate glass. Partially crystallized specimens comprising ZnWO_4_ as the crystalline phase were obtained from the compositions having 15 and 20 mol % WO_3_. 

As a continuation of our previous studies, mentioned above, in this work, we investigate the effect of the addition of smaller amounts of WO_3_ (up to 5 mol %) on the structure and luminescent properties of ZnO-B_2_O_3_ glass doped with 0.5 mol % Eu_2_O_3_ by using Raman spectroscopy, differential scanning calorimetry and photoluminescent spectroscopy. The aim is to find the optimal glass composition, ensuring the most appropriate structure for accommodating the active rare earth ion, that will improve its luminescence properties.

## 2. Results

### 2.1. Thermal Analysis

Bulk transparent glasses were obtained from the nominal compositions in mol % of 50ZnO:(50 − x)B_2_O_3_:0.5Eu_2_O_3_:xWO_3_, x = 0, 1, 3, 5. Glass samples having 10 mol % WO_3_ (50ZnO:40:B_2_O_3_:0.5Eu_2_O_3_:10WO_3_), previously reported by us in ref. [13], were prepared and investigated again in the present work to compare their structure and luminescence behavior with those of glasses having lower WO_3_ content. Having in mind the high vapor pressure of WO_3_ oxide, which is the doping component, we accept that its actual concentration does not change significantly from the nominal one after the melting of the compositions. The X-ray diffractograms of the investigated glasses, along with the photographic images of the samples, are shown in references [13,14]. All investigated samples were X-ray amorphous. The prepared WO_3_--free glass was colorless, while WO_3_--containing glasses were brownish, due to the presence of some amount of Eu^2+^ ions, established by EPR measurements in ref. [13].

**Figure 1:**
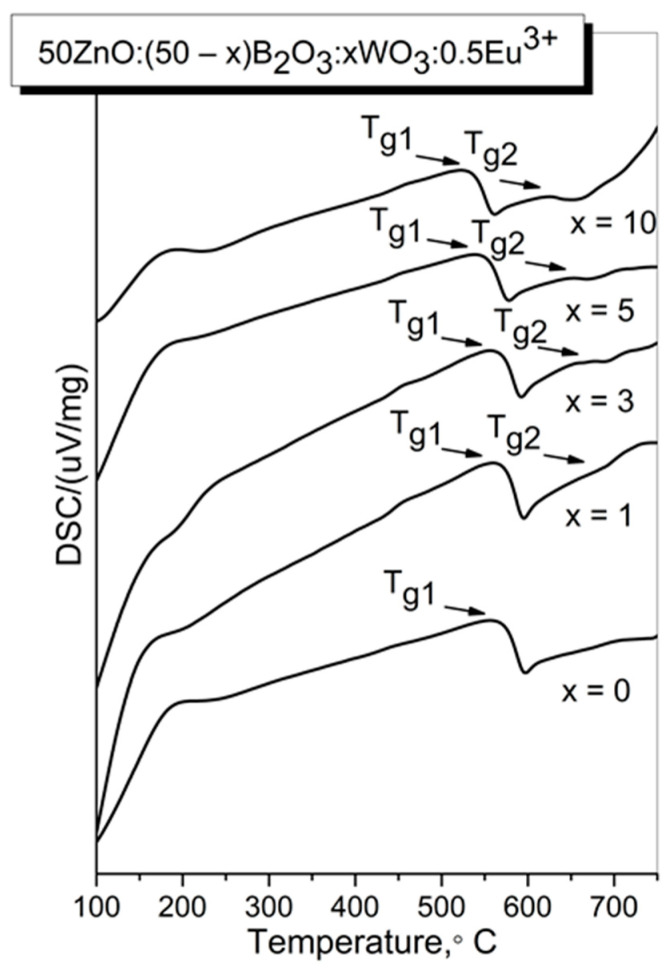
DSC curves of the obtained glasses.

50ZnO:(50 − x)B_2_O_3_:xWO_3_:0.5Eu_2_O_3_, (x = 0, 1, 3, 5 and 10 mol %) glasses have been also investigated by DSC analysis in order to obtain information for some thermal parameters and for structural changes that take place due to the compositional changes [15]. The glass transition temperature, T_g_, has been determined, since it is connected with both the strength of inter-atomic bonds and glass network connectivity. A higher T_g_ corresponds to a more rigid structure, whereas the glasses having a loose-packed structure have a lower T_g_ [16,17,18]. Figure 1 compares the DSC curves of the glasses investigated in this work. Two humps, corresponding to the two glass transition temperatures T_g1_ and T_g2_, are observed. Their values are listed in Table 1. The presence of two glass transition effects is connected with the formation of two amorphous phases with different compositions in the investigated glasses. 

As one can see, the addition of WO_3_ into zinc borate glass causes a slight decrease in the glass transition temperature values. Having in mind our previous IR data and as well as the established values of structurally sensitive physical parameters [14], we explain the observed reduction in T_g1_ and T_g2_ as a result of increasing non-bridging atoms with the addition of WO_3_.

The values of T_g1_ and T_g2_ were correlated with average single-bond enthalpy, *E_B_*, of glasses using the following relationship proposed in ref. [19]:

$$E_{B}=\frac{E_{50Zn\text{-}O}+E_{(50-x)B\text{-}O}+E_{xW\text{-}O}+E_{0.5Eu\text{-}O}\;}{100}$$

where *E_W-O_*; *E_B-O_*, *E_Eu-O_* and *E_Zn-O_* are the bond dissociation energies for the single bonds: W-O; B-O, Eu-O and Zn-O, respectively [20]. *E_B_* values gradually decrease with WO_3_ loading because of the gradual replacement of stronger B-O bonds with higher bond dissociation energy (808 kJ mol^−1^) by weaker W-O bonds with smaller bond dissociation energy (653 kJ mol^−1^) [20]. 

### 2.2. Structural Analysis

In our previous studies, we have investigated the structure of these glasses by applying IR spectroscopy [13,14]. The analysis revealed the presence of several borate structural units in the glass network as [BØ_2_O]^−^ and [BØ_4_]^−^ metaborate groups, and pyroborate [B_2_O_5_]^4−^ entities. WO_3_ modifies the borate network, resulting in a decrease in [BØ_4_]^−^ units and the favoring of the formation of pyroborate dimers, [B_2_O_5_]^4−^. Tungstate ions incorporate into base zinc borate glass as tetrahedral [WO_4_]^2−^ groups, and octahedral [WØ_4_O_2_]^2−^ species with four bridging and two non-bridging oxygen atoms. The densities of these glasses have been measured, and on this basis, several structurally sensitive parameters such as molar volume (V_m_), oxygen volume (V_o_) and oxygen packing density (OPD) have been also established and are reported in refs. [13,14]. Their values also showed the depolymerization of the glass structure of the base zinc borate glass (increasing NBOs) when WO_3_ is added. 

**Figure 2:**
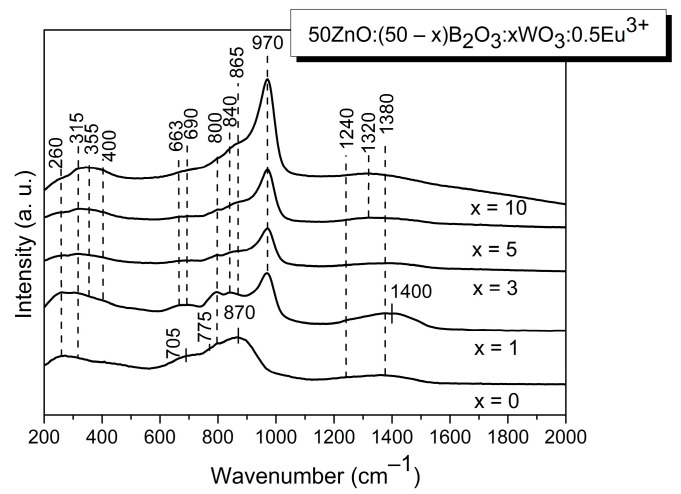
Raman spectra of the obtained glasses.

In the present study, we have used, additionally, Raman spectroscopy in order to obtain more complete structural information about the investigated glasses. The Raman spectra obtained are shown in Figure 2. The Raman spectrum of the base zinc borate glass, without WO_3_ (Figure 2; x = 0), is in good agreement with what has been reported by other authors for similar compositions [21,22,23]. It contains a strong and broad band centered at about 870 cm^−^^1^, bands at 800 cm^−^^1^ and 775 cm^−^^1^, a broad shoulder at about 705 cm^−^^1^, features at about 260 cm^−^^1^ and 315 cm^−^^1^ and a high-frequency envelope from about 1200 to 1550 cm^−1^. 

The most prominent band, at 870 cm^−1^, observed only in the Raman spectrum of glass x = 0, is due to the symmetric stretching of B-O-B bridges in pyroborate dimers, [B_2_O_5_]^4−^ [21,22,23]. The band at 800 cm^−^^1^ is connected with the ring breathing of the boroxol rings, while the not-well-resolved band at 775 cm^−1^ signals the presence of six-membered borate rings consisting of two [BO^0^] triangles and one tetrahedral unit [BO_4_]^−^ (i.e., triborate rings) [21,22,23]. The broad shoulder at about 705 cm^−1^ contains contributions of at least four borate arrangements: deformation modes of metaborate chains, [BØ_2_O]^−^ (Ø = bridging oxygen, O^−^ = nonbridging oxygen), in-plane and out-of-plane bending modes of both polymerized (BØ^0^) species and isolated orthoborate units (BO_3_)^3−^, and bending of the B-O-B connection in the pyroborate dimers, [B_2_O_5_]^4−^) [21,22,23]. The higher-frequency activity in the 1200 to 1550 cm^−1^ range is connected with the stretching vibrations of B-O^−^ bonds, involving non-bridging oxygen (NBO) in the pyroborate dimers (1240 cm^−^^1^), and in metaborate triangular units, BØ_2_O^−^ (1380 cm^−^^1^) [21,22,23]. The lower-frequency features at 260 and 315 cm^−1^ are related to the Zn-O vibrations and Eu-O vibrations, respectively [13]. The addition of WO_3_ influences the Raman spectrum of the glass x = 0 considerably. A new and strong band at 970 cm^−^^1^ appears, due to the ν_1_ symmetric stretching mode of isolated [WO_4_]^2−^ tetrahedra, charge balanced by Zn^2+^ and Eu^3+^ ions [13]. Another band, characteristic of the [WO_4_]^2−^ tetrahedral units, is the band at 800 cm^−^^1^, which is due to the asymmetric stretching ν_3_ mode of tetrahedral [WO_4_]^2−^ groups. This band significantly overlaps with the ring breathing mode of the boroxol rings. The band at 775 cm^−^^1^, due to the six-membered borate rings with one [BO_4_]^-^ unit present in the spectrum of WO_3_-free zinc borate glass (Figure 2, x = 0), disappears in the spectra of glasses containing WO_3_, indicating the destruction of these structural groups when WO_3_ is added. Thus, depolymerization of the borate oxygen network took place, which is in agreement with the previously reported IR data [13,14]. The low-frequency band at 315 cm^−^^1^, attributed to the Eu-O vibration in the glass x = 0, overlaps with the ν_2_ [WO_4_]^2−^ mode, which explains its increasing intensity upon WO_3_ loading. Another new low-frequency band at 355 cm^−^^1^ is attributed to the ν_4_ bending mode of the [WO_4_]^2−^ tetrahedra [13]. In addition to tungstate tetrahedral groups, there are also tungstate octahedral [WØ_4_O_2_]^2−^ species with four bridging and two non-bridging oxygen atoms in the structure of the studied glasses, which by edge-sharing form [W_2_O_8_^4−^] polymeric anions [13]. These tungstate species are charge balanced by Zn^2+^ and Eu^3+^ cations [13]. The presence of several new bands in the spectra of WO_3_-containing glasses at 840 cm^−^^1^, 865 cm^−^^1^, 663 cm^−^^1^, 690 cm^−^^1^ and 400 cm^−^^1^ can be discussed in terms of the vibrations of the WO_2_ terminal units and [W_2_O_4_]_n_ chains of the [W_2_O_8_^4-^]_n_ anions, as was reported in the literature for the ZnWO_4_ compound [13,24]. In particular, Raman bands at 840 and at 865 cm^−^^1^ are connected with ν_as_(WO_2_) and ν_s_(WO_2_) vibrations of terminal WO_2_ units. The bands at 663, 690 cm^−^^1^ and 400 cm^−^^1^ are related to ν_as_[W_2_O_4_]_n_ and ν_s_[W_2_O_4_]_n_ vibrations, which involve mainly two-oxygen bridges (W_2_O_2_) of the chain structure [W_2_O_4_]_n_ [13]. On the other hand, the band at 840 cm^−^^1^ in the spectra of WO_3_-containing glasses can be also connected with the symmetric B-O-B stretching of pyroborate dimmers, [B_2_O_5_]^4−^ [21,23]. Because of its complex character, the band growth with increasing WO_3_ loads observed is not possible to explain in a straightforward manner. The Raman activity in the high-frequency region, 1200–1550 cm^−^^1^*,* is connected with the vibration of B-O-containing borate arrangements. More particularly, the higher-frequency activity at 1240 cm^−1^ reflects the symmetric stretch of boron-non-bridging oxygen bonds, ν(B-O^−^) of the pyroborate dimers, while the other two features at 1380 and 1400 cm^−1^ are due to the B-O^−^ stretching in metaborate triangular units, [BØ_2_O]^−^ [21,22,23]. There is a red shift in the frequency of the band at 1380 cm^−^^1^ to 1320 cm^−^^1^ in the spectra of glasses x = 5 and x = 10. As is reported in ref. [21], the frequency of the boron oxygen stretching in trigonal metaborate units is highly sensitive to the surrounding environment, i.e., the degree of the covalency of the non-bridging oxygen with the charge-balancing cations. The red shift observed indicates that in the glasses having higher WO_3_ content (5 and 10 mol %), [BØ_2_O]^−^ entities are charge balanced mainly by Zn^2+^ and Eu^3+^ ions. The proposed band assignments are summarized in Table 2.

### 2.3. Optical Transmission Spectra

**Figure 3:**
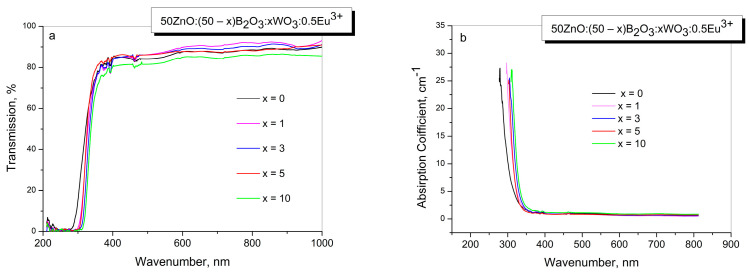
Optical transmission spectra at room temperature, (**a**), and absorption coefficient in the range of 150 nm–900 nm, (**b**) for studied glasses.

Figure 3a,b presents the optical transmission spectra and absorption coefficient data of the investigated glasses. As is seen from Figure 3a, glasses are characterized as having good transmission in the visible region of the electromagnetic spectrum at around 80%; this slightly decreases with the WO_3_ loading. The changes in the optical transmission observed are due to the low coloration of the glasses containing WO_3_. In addition, the absence of any absorption bands in the visible range indicates that there are no tungstate ions in the lower-than-W^+6^ valance state, since the reduced W^+5^ and W^+4^ ions produce very intensive absorption bands in the visible range due to d-d transition [25]. 

Two weak absorption bands at 392 and 463 nm are observed, corresponding to the f-f transitions of Eu^3+^ ions between the ground and the excited states. We have calculated the absorption coefficient (*α*) using the following equation:

$$\alpha =\left\{\ln\left(\frac{100}{T}\right)\right\}/d$$

where *T* is the percentage transmission and *d* is the thickness of the glass. The absorption coefficients versus wavelength spectra are presented in Figure 3b. The maximum absorption values of the glasses increase with the increase in WO_3_ content and vary between 280 and 311 nm.

### 2.4. Luminescent Properties

**Figure 4:**
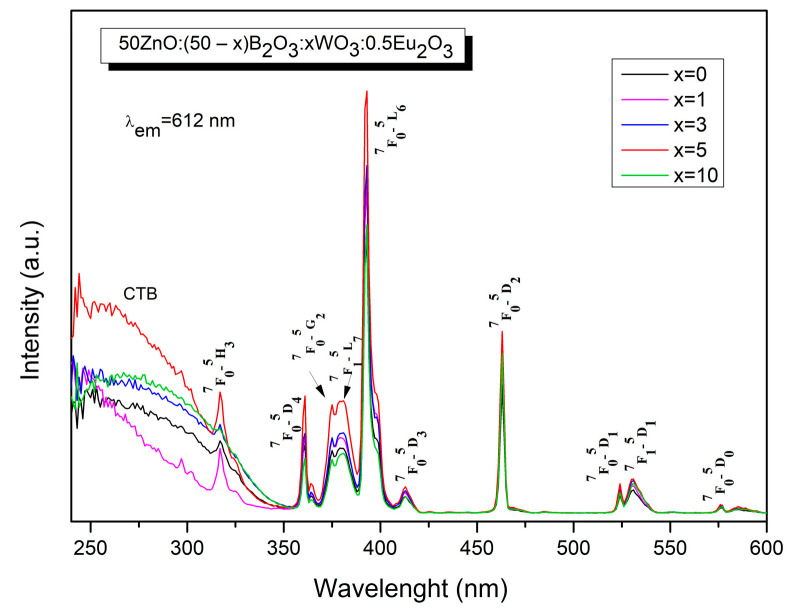
Excitation spectra of 50ZnO:(50 − x)B_2_O_3_:xWO_3_:0.5Eu_2_O_3_ (x = 0, 1, 3, 5 and 10) glasses.

The optical properties of the obtained glasses were further studied. The excitation spectra of 50ZnO:(50 − x)B_2_O_3_:xWO_3_:0.5Eu_2_O_3_ (x = 0, 1, 3, 5 and 10) glasses, shown in Figure 4, are recorded at room temperature, monitoring the most prominent Eu^3+^ emission at 612 nm (^5^D_0_ → ^7^F_2_ transition). The spectra consist of two distinct parts. The first part is composed of a broad band in the spectral range from 250 nm to 350 nm due to the ligand-to-metal charge transfer transitions (LMCT) of O^2−^ → W^6+^ [26] and O^2−^ → Zn^2+^ [27] inside the WO_n_ (WO_n_ = WO_4_ and WO_6_) and ZnO_n_ (ZnO_n_ = ZnO_4_) host absorbing groups, respectively, and from the oxygen 2p orbital to the empty 4f orbital of europium (O^2−^→ Eu^3+^) [28,29,30,31]. Additionally, several sharp intra-configurational 4f → 4f transitions of Eu^3+^ can be observed in the spectra at 317 nm, 375 nm, 380 nm, 392 nm, 413 nm, 463 nm, 524 nm, 531 nm and 576 nm due to ^7^F_0_ → ^5^H_3_, ^7^F_0_ → ^5^D_4_, ^7^F_0_ → ^5^G_2_, ^7^F_1_ → ^5^L_7_, ^7^F_0_ → ^5^L_6_, ^7^F_0_ → ^5^D_3_, ^7^F_0_ → ^5^D_2_, ^7^F_0_ → ^5^D_1_, ^7^F_1_ → ^5^D_1_, ^7^F_0_ → ^5^D_0_, respectively [32]. The ^7^F_0_ → ^5^H_3_ excitation peak at 317 nm is superposed over the broad charge transfer band. The strongest excitation lines are observed at 392 nm and 463 nm, assigned to the ^7^F_0_ → ^5^L_6_ and ^7^F_0_ → ^5^D_2_ transitions in the near-UV and visible blue regions. These wavelengths are suitable for excitation with commercial near-ultraviolet light-emitting diodes (LEDs) (250–400 nm) and blue LED chips (430–470 nm). 

**Figure 5:**
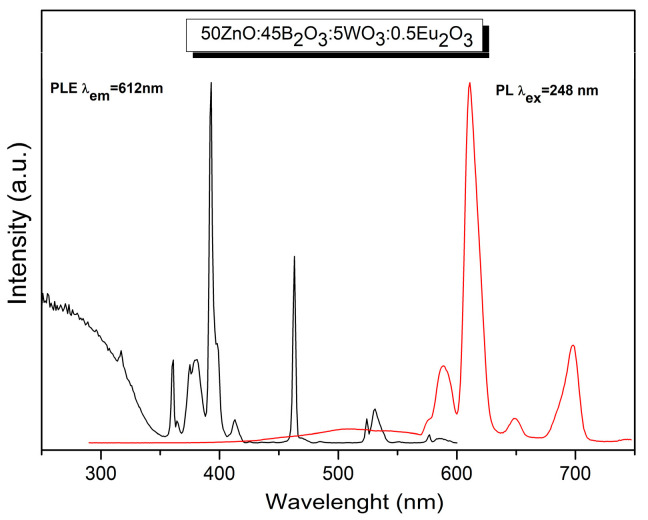
Excitation (black line) and emission (red line) spectra of 0.5 mol % Eu^3+^-doped 50ZnO:45B_2_O_3_:5WO_3_ glass.

As can be seen from Figure 4, with increasing concentration of WO_3_ in the glass composition, the intensity of Eu^3+^ f-f transitions increases for up to 5 mol % WO_3_. A further increase in WO_3_ leads to a decrease in the excitation intensity. Accordingly, the addition of WO_3_ into Eu^3+^-doped 50ZnO:50B_2_O_3_ host glass up to 5 mol % is beneficial to achieve good excitation, because as a rule, Eu^3+^ excitation peaks are characterized by low intensity due to the parity-forbidden law. The appearance of absorption of the host matrix, when monitoring the Eu^3+^ emission at 612 nm, has been shown to play an important role in the enhancement of the rare earth emission intensity through the occurrence of non-radiative energy transfer, in particular from WO_n_ and ZnO_n_ structural polyhedra to the Eu^3+^ ion [13,14,26,33,34,35,36,37,38]. This process is known as “host sensitized” energy transfer. Additionally, according to data from the literature, the 350 nm–700 nm region is registered to be the broad emission band (red line, Figure 5) of WO_3_ [39] and ZnO [27]. In the same spectral region are also located the excitation bands of Eu^3+^ (black line) [32]. The other requirement for energy transfer is the overlap of the host group emission and the excitation levels of the active ion. As can be seen from Figure 5, in our case, this condition is satisfied.

**Figure 6:**
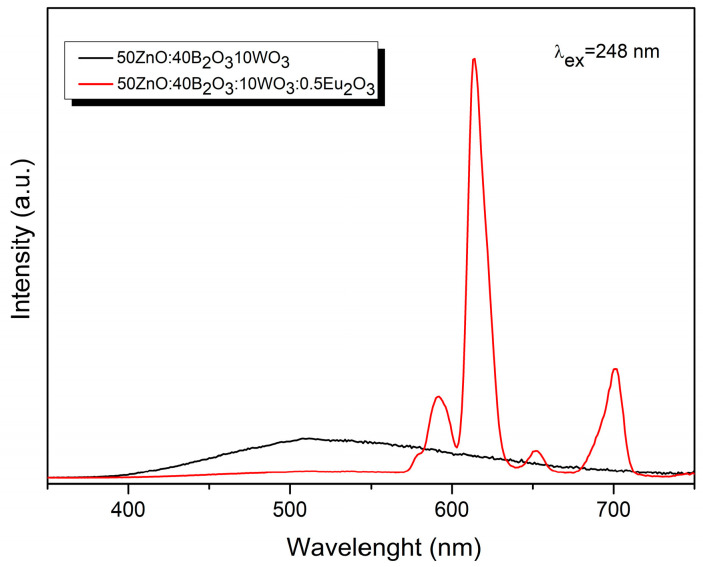
Emission spectra of host matrix 50ZnO:40B_2_O_3_:10WO_3_ (black line) and Eu^3+^-doped 50ZnO:40B_2_O_3_:10WO_3_ glass (red line) at 248 nm excitation.

**Figure 7:**
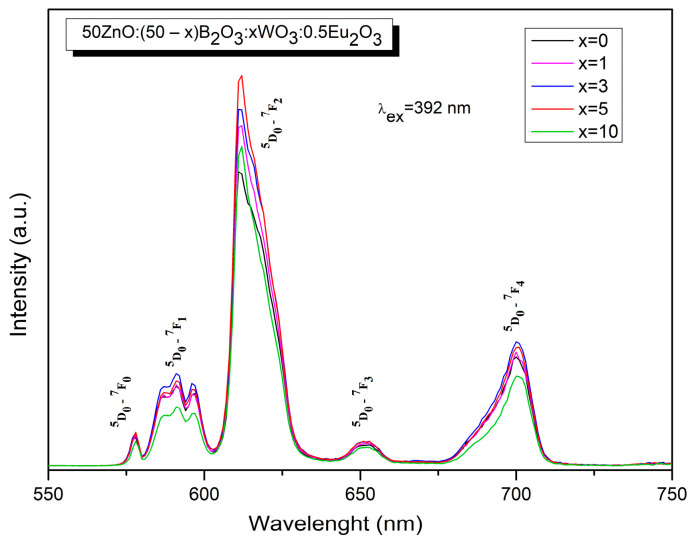
PL emission spectra of glasses 50ZnO:(50 − x)B_2_O_3_:xWO_3_:0.5Eu_2_O_3_ (x = 0, 1, 3, 5 and 10).

In Figure 6, it can be seen the emission spectra of pure and Eu^3+^-doped 50ZnO:40B_2_O_3_:10WO_3_ glass matrix were acquired upon excitation at the maximum value of the host charge transfer band at 248 nm (WO_n_ and ZnO_n_ absorbing groups). The observed decrease in the emission of the host absorbing groups in Eu^3+^-doped glass (red line), as compared to pure host emission (black line) [27,39], evidences that energy transfer has occurred. In other words, the energy absorbed by tungstate and zincate groups is further transferred non-radiatively to the active Eu^3+^ ion. The results obtained above also imply that Eu^3+^ and the WO_n_ and ZnO_n_ groups are closely coordinated in the structure [33]. It is known that the probability of energy transfer increases when the host absorbing groups, in our case WO_n_ or ZnO_n_ and the Eu^3+^ active ion, are nearest neighbors in the structure [40]. The emission spectra of Eu^3+^-doped glasses are also obtained by monitoring the ^7^F_0_ → ^5^L_6_ transition at 392 nm (Figure 7). 

The characteristic intra-configurational transition of Eu^3+ 5^D_0_→^7^F_J_, where J = 0, 1, 2, 3, 4 at 578 nm, 591 nm, 642 nm, 652 nm and 700 nm, appeared in the spectra [32]. The addition of WO_3_ into the glass composition leads to an increase in the emission intensity for up to 5 mol %. At 10 mol % WO_3_, a luminous quenching is observed in the spectra. Among the Eu^3+^ sharp emission bands, the most intense one, located at 612 nm, was allowed by the electric dipole and was sensitive to the changes in the surrounding ^5^D_0_→^7^F_2_ transition (red color of emission), followed by the magnetic dipole, which allowed the ^5^D_0_→^7^F_1_ transition (orange emitting color), which is insensitive to the coordination environment around Eu^3+^ ions. The ratio between these emissions, known as the asymmetric ratio R, can reveal the degree of asymmetry in the local environment around the Eu^3+^ and the strength of the Eu–O covalence for various Eu^3+^-doped compounds. The lower the value of the asymmetry parameter, the higher the symmetry around the active ion, and the lower the Eu–O covalency and emission intensity. The increase in R value is due to the increase in asymmetry and covalency between the Eu^3+^ ion and the ligands [41]. The R values of the synthesized glasses are listed in Table 3 along with other data reported in the literature about Eu^3+^-doped oxide glasses [6,13,34,42,43,44,45,46,47,48]. The relatively higher values compared to other reported Eu^3+^-doped oxide glasses indicate that Eu^3+^ occupies crystallographic sites with low symmetry and also provide evidence of the high Eu^3+^-O^2−^ covalency. The highest obtained R value of 5.82, attributed to 50ZnO:45B_2_O_3_:5WO_3_:0.5Eu_2_O_3_, is an indication of the highest emission intensity. Additionally, the Eu^3+ 5^D_0_→^7^F_1_ transition splits into three emission peaks, centered at 586 nm, 591 nm and 596 nm. This effect probably arises from crystal field splitting, which causes a single transition to produce multiple emission peaks [49]. Furthermore, the appearance of the ^5^D_0_→^7^F_0_ transition, which is sensitive to the crystal field and is forbidden based on the standard Judd–Ofelt theory, indicates that the Eu^3+^ ion occupies non-centrosymmetric sites with C_2ν_, C_n_ or C_s_ symmetry [50].

#### CIE Color Coordinates and CCT (K) Values

**Figure 8:**
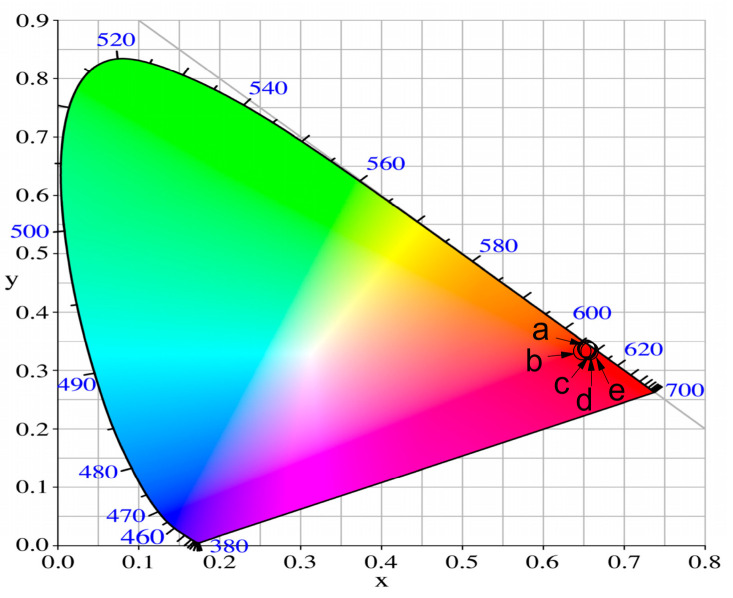
CIE chromaticity diagram of the 50ZnO:(50 − x)B_2_O_3_:xWO_3_:0.5Eu_2_O_3_ (a) x = 0, (b) x = 1, (c) x = 3, (d) x = 5, (e) x = 10 glasses.

To better understand the luminescent behavior and the actual color of emissions, the standard Commission Internationale de l’Eclairage (CIE) 1931 chromaticity diagram was used [51]. The color chromaticity coordinates of the synthesized glasses are calculated from the PL spectra (Figure 7), using SpectraChroma software, Version 1.0.1 (CIE coordinate calculator) [52] and are shown at Figure 8. The obtained values of glasses with different WO_3_ content, enlisted at Table 4, are almost similar and are very close to the CIE coordinate of standard red light (0.67, 0.33) and to the color coordinates of the commercial red phosphor Y_2_O_2_S:Eu^3+^ (0.658; 0.340) [53].

The values of correlated color temperature (CCT) were calculated by the McCamy empirical expression [54]:

$$CCT=-449n^{3}+3525n^{2}-6823n+5520.33;$$

$$n=\frac{\left(x-x_{e}\right)}{\left(y-y_{e}\right)}$$

Here, *x*_e_ = 0.332, *y*_e_ = 0.186 are the epicenter coordinates and *x* and *y* are the calculated color chromaticity coordinates. In our case, the calculated CCT values shown in Table 4 are in the range of 2270.30 K ÷ 2439.74 K, and the Eu^3+^-doped glasses can be referred to as warm light-emitting materials. 

## 3. Discussion

Raman analysis revealed that tungstate ions incorporate into the base zinc borate glass as tetrahedral [WO_4_]^2−^ groups, and octahedral [WØ_4_O_2_]^2−^ species with four bridging and two non-bridging oxygen atoms. There are also metaborate, [BØ_2_O]^−^ and pyroborate units, [B_2_O_5_]^4−^ in the glass networks. Tungstate, metaborate and pyroborate units are charge balanced by Zn^2+^ and Eu^3+^ ions via Zn-O-W, Zn-O-B, Eu-O-W and Eu-O-B bonding. As it was proved in our previous paper, W-O-B bonding is not possible [25]. Thus, the investigated glasses are characterized by a heterogeneous structure, because in the main zinc borate glass network, regions rich in tungsten are formed. Structural heterogeneity in these glasses increases with increasing WO_3_ content, as the number of tungstate structural units, as well as Zn-O-W bonds, increase. This observation has been also confirmed by DCS analysis, wherein two glass transition effects have been noted in the DSC curve of glasses, due to the presence of two amorphous phases with different compositions. On the other hand, the addition of WO_3_ into the base zinc borate glass causes depolymerization of the borate oxygen network, i.e., increasing the number of non-bridging oxygen atoms. In this way, with increasing WO_3_ concentrations, the structural disorder in the amorphous network increases, and also, a more loosened glass structure is formed. The established structural features of the investigated glasses determine their good emission properties. All glasses are characterized by a high-intensity red luminescence band at 612 nm corresponding to the forced electric dipole transition (ED) ^5^D_0_ → ^7^F_2_, which is stronger for the WO_3_-containing glasses as compared with the WO_3_-free glass. The addition of WO_3_ to the base zinc borate glass improves the Eu^3+^’s luminescence properties, which can be explained by the presence of both borate and tungstate units in the active ions’ surroundings, which increases the Eu^3+^’s site asymmetry and hence the Eu^3+^’s emission intensity. Also, the presence of tungstate groups around Eu^3+^ ions ensures an occurrence of non-radiative energy transfer from tungstate units to the active ions, which additionally improves the Eu^3+^’s luminescence behavior. The observed maximum of Eu^3+^ emission for glass x = 5 shows that this glass composition ensures the most appropriate glass structure for accommodating the active ion. With a further increase in WO_3_ content of 10 mol %, glass network heterogeneity is too high to promote the good distribution of the rare earth ions in the matrix, and, thus, the emission properties of europium are reduced. On the other hand, with the incorporation of a higher amount of WO_3_, the average distance between tungstate groups would be decreased, which would make the energy transfer between them more sufficient and, thus less energy would be expected to be transferred to the Eu^3+^ ions [55]. 

## 4. Materials and Methods

Glasses with nominal compositions 50ZnO:(50 − x)B_2_O_3_:xWO_3_:0.5Eu_2_O_3_, (x = 0, 1, 3, 5 and 10 mol %) were obtained by applying the conventional melt-quenching method, using commercial powders of reagent-grade WO_3_ (Merck KGaA, Darmstadt, Germany), ZnO (Merck KGaA, Amsterdam, The Netherlands), H_3_BO_3_ (SIGMA-ALDRICH, St. Louis, MO, USA), and Eu_2_O_3_ (SIGMA-ALDRICH, St. Louis, MO, USA) as starting materials. The details of glass synthesis were given in refs. [13,14]. The glass transition (T_g_) temperatures of the glasses were determined by differential scanning calorimetry (DSC) using a Netzsch 404 Pegasus instrument, 2021 Selb, Germany, at a heating rate of 10 K/min in an Ar flow of 10 mL/s, using corundum crucibles with lids. Optical transmission spectra at room temperature for the glasses were measured by a spectrometer (Ocean Optics, HR 4000, Duiven, 2010, The Netherlands) using a UV LED light source at 385 nm. Photoluminescence (PL) excitation and emission spectra at room temperature for all glasses were measured with a Spectrofluorometer FluoroLog3-22, 2014 (Horiba JobinYvon, Longjumeau, France). Raman spectra were recorded with a Raman spectrometer (Delta NU, Advantage NIR 785 nm, 2010, Midland, ON, Canada).

## 5. Conclusions

The present investigation demonstrates the relationship between the host glass structure and the optical properties of Eu^3+^-doped glasses 50ZnO:(50 − x)B_2_O_3_:xWO_3_, x = 0 1, 3, 5 and 10 mol %. The result indicates that the addition of WO_3_ of up to 5 mol % to the base zinc borate glass improves the luminescence intensity of doped rare earth ions, which is attributed to the formation of a more disordered glass network where Eu^3+^ ions are surrounded by both borate and tungstate units, which ensures a highly asymmetric local structure around Eu^3+^ ions sites, accordingly yielding a strong red emission of the active ions. A further increase in the tungsten content leads to luminescent quenching. All findings obtained here are favorable for the elaboration of novel red-emitting glass materials.

## Figures and Tables

**Table 1:** Values of glass transition temperatures (T_g_) and average single-bond enthalpy (E_B_) of the investigated glasses.

Sample ID	T_g1_/°C	T_g2_/°C	E_B_/kJ mol^−1^
x = 0	570 (843 K)	-	548
x = 1	568 (841 K)	688 (961 K)	546
x = 3	565 (838 K)	680 (953 K)	543
x = 5	543 (816 K)	653 (926 K)	540
x = 10	532 (805 K)	626 (899 K)	532

**Table 2:** Peak positions in the Raman spectra of glasses 50ZnO:(50 − x)B_2_O_3_:0.5Eu_2_O_3_:xWO_3_, (x = 0, 1, 3, 5 and 10 mol %) and their assignments.

Peak Positions	Assignments	Ref.
260	ν (Zn-O)	[21,22]
315	ν (Eu-O) + ν_2_ [WO_4_]^2^	[13]
355	ν_4_ [WO_4_]^2−^	[13]
400	ν_s_[W_2_O_4_]_n_	[13]
663	ν_as_[W_2_O_4_]_n_	[13]
690	ν_as_[W_2_O_4_]_n_	[13]
705	δ[BØ_2_O]^−^ + in-plane and out-of-plane bending modes of both polymerized (BØ^0^) species and isolated orthoborate units (BO_3_)^3-^+ bending of the B-O-B connection in the pyroborate dimers, [B_2_O_5_]^4−^	[21,22,23]
800	ring breathing of the boroxol rings+ ν_3_[WO_4_]^2−^	[13,21,22,23]
840	ν_as_(WO_2_) + B-O-B stretching of [B_2_O_5_]^4−^	[13]
865	ν_s_(WO_2_)	[13]
870	B-O-B bridges in pyroborate dimers, [B_2_O_5_]^4−^	[21,22,23]
970	ν_1_[WO_4_]^2−^	[13]
1240	B-O^−^ stretch in pyroborate units	[21,22,23]
1380–1320	B-O^−^ stretch in metaborate units	[21,22,23]
1400	B-O^−^ stretch in metaborate units	[21,22,23]

**Table 3:** Values of the relative intensity ratio (R) for 50ZnO:(50 − x)B_2_O_3_:xWO_3_:0.5Eu_2_O_3_ (x = 0, 1, 3, 5 and 10) glasses.

Glass Composition	Relative Luminescent Intensity Ratio, R	Reference
50ZnO:50B_2_O_3_:0.5Eu_2_O_3_	4.34	Current work
50ZnO:49B_2_O_3_:1WO_3_:0.5Eu_2_O_3_	5.67	Current work
50ZnO:47B_2_O_3_:3WO_3_:0.5Eu_2_O_3_	5.71	Current work
50ZnO:45B_2_O_3_:5WO_3_:0.5Eu_2_O_3_	5.82	Current work
50ZnO:40B_2_O_3_:10WO_3_:0.5Eu_2_O_3_	5.57	Current work + [13]
50ZnO:(50 − x)B_2_O_3_: xNb_2_O_5_:0.5Eu_2_O_3_:, x = 0, 1, 3 and 5 mol %	4.31–5.16	[42]
50ZnO:40B_2_O_3_:10WO_3_:xEu_2_O_3_ (0 ≤ x ≤ 10)	4.54÷5.77	[13]
50ZnO:40B_2_O_3_:5WO_3_:5Nb_2_O_5_:xEu_2_O_3_ (0 ≤ x ≤ 10)	5.09÷5.76	[34]
4ZnO:3B_2_O_3_ 0.5–2.5 mol % Eu_2_O_3_	2.74–3.94	[43]
60TeO_2_:39ZnO:1Eu_2_O_3_	3.25	[44]
60TeO_2_:20ZnO:19LiF:1Eu_2_O_3_	3.70	[44]
60TeO_2_:19ZnO:10Na_2_O:10Li_2_O:1Eu_2_O_3_	3.73	[44]
60ZnO:20B_2_O_3_:(20 *−* x)SiO_2−__x_Eu_2_O_3_ (x = 0 and 1)	3.166	[6]
[{(TeO_2_)_0.7_:(B_2_O_3_)_0.3_}_0.7_:(ZnO)_0.3_]_(1−y)_(Eu_2_O_3_)_y_ with 0.01 = y ≤ 0.05	2.15–3.08	[45]
59.5Li_2_O:39.5B_2_O_3_:1Eu_2_O_3_	4.18	[46]
19.5Na_2_O:20MgO:59.5SiO_2_:1Eu_2_O_3_	4.43	[47]
(15 − x)WO_3_–5Al_2_O_3_–80TeO_2_–xEu_2_O_3_; x = 0.1÷5 mol %	5.4÷6	[48]

**Table 4:** CIE chromaticity coordinates and correlated color temperatures (CCTs, K) of 50ZnO:(50 − x)B_2_O_3_:xWO_3_:0.5Eu_2_O_3_ (x = 0, 1, 3, 5 and 10).

Glass Composition	Chromaticity Coordinates (x, y)	CCT (K)
50ZnO:50B_2_O_3_:0.5Eu_2_O_3_	0.645, 0.346	2301.26
50ZnO:49B_2_O_3_:1WO_3_:0.5Eu_2_O_3_	0.651, 0.348	2324.59
50ZnO:47B_2_O_3_:3WO_3_:0.5Eu_2_O_3_	0.650, 0.350	2270.30
50ZnO:45B_2_O_3_:5WO_3_:0.5Eu_2_O_3_	0.652, 0.347	2359.79
50ZnO:40B_2_O_3_:10WO_3_:0.5Eu_2_O_3_	0.654, 0.345	2439.74
NTSC standard for red phosphors	0.670, 0.330	
Y_2_O_2_S:Eu^3+^	0.658, 0.340	

## Data Availability

Data are contained within the article.
